# ENPP1 enzyme replacement therapy improves blood pressure and cardiovascular function in a mouse model of generalized arterial calcification of infancy

**DOI:** 10.1242/dmm.035691

**Published:** 2018-10-08

**Authors:** Tayeba Khan, Kerstin W. Sinkevicius, Sylvia Vong, Arlen Avakian, Markley C. Leavitt, Hunter Malanson, Andre Marozsan, Kim L. Askew

**Affiliations:** 1 Alexion Pharmaceuticals, Lexington, MA 02421, USA; 2 Alexion Pharmaceuticals, New Haven, CT 06510, USA

**Keywords:** Enpp1, GACI, Vascular calcification, Enzyme replacement therapy, Hypertension

## Abstract

Generalized arterial calcification of infancy (GACI) is a rare, life-threatening disorder caused by loss-of-function mutations in the gene encoding ectonucleotide pyrophosphatase phosphodiesterase 1 (*ENPP1*), which normally hydrolyzes extracellular ATP into AMP and pyrophosphate (PP_i_). The disease is characterized by extensive arterial calcification and stenosis of large- and medium-sized vessels, leading to vascular-related complications of hypertension and heart failure. There is currently no effective treatment available, but bisphosphonates – nonhydrolyzable PP_i_ analogs – are being used off-label to reduce arterial calcification, although this has no reported impact on the hypertension and cardiac dysfunction features of GACI. In this study, the efficacy of a recombinant human ENPP1 protein therapeutic (rhENPP1) was tested in *Enpp1^asj-2J^* homozygous mice (*Asj-2J* or *Asj-2J* hom), a model previously described to show extensive mineralization in the arterial vasculature, similar to GACI patients. In a disease prevention study, *Asj-2J* mice treated with rhENPP1 for 3 weeks showed >95% reduction in aorta calcification. Terminal hemodynamics and echocardiography imaging of *Asj-2J* mice also revealed that a 6-week rhENPP1 treatment normalized elevated arterial and left ventricular pressure, which translated into significant improvements in myocardial compliance, contractility, heart workload and global cardiovascular efficiency. This study suggests that ENPP1 enzyme replacement therapy could be a more effective GACI therapeutic than bisphosphonates, treating not just the vascular calcification, but also the hypertension that eventually leads to cardiac failure in GACI patients.

## Abstract (precis)

**Summary:** ENPP1 enzyme replacement therapy can have important implications for generalized arterial calcification of infancy by treating both vascular calcification and hypertension, which are the leading causes of cardiac failure and mortality in patients.

## INTRODUCTION

Generalized arterial calcification of infancy (GACI) is a severe, rare disease characterized by excessive calcification and stenosis of large- and medium-sized arteries. Arterial stenosis occurs due to severe myointimal proliferation and reduced vascular elasticity, and can eventually lead to hypertension, myocardial ischemia and heart failure. Other hallmarks of the disease include cardiomegaly, respiratory distress and cyanosis. Affected arteries in GACI include the aorta, coronary arteries, pulmonary arteries and renal arteries (Chong and Hutchins, 2008; Rutsch et al., 2008). Histological examination of affected arteries often shows calcium deposition in the internal elastic lamina and fibrointimal hyperplasia, both of which can contribute to luminal narrowing (Moran, 1975; Morton, 1978; Witzleben, 1970).

Approximately 70% of GACI cases are associated with loss-of-function mutations in *ENPP1*, an ectonucleotide pyrophosphatase/phosphodiesterase 1. ENPP1 is a type II extracellular transmembrane glycoprotein expressed in a range of cell types, including osteoblasts, osteoclasts, chondrocytes and vascular smooth muscle cells (Goding et al., 1998; Hajjawi et al., 2014; Hessle et al., 2002; Prosdocimo et al., 2009). The main function of ENPP1 is to hydrolyze extracellular adenosine triphosphate (ATP) into adenosine monophosphate (AMP) and pyrophosphate (PP_i_), a potent physiological inhibitor of hydroxyapatite formation and vascular calcification. Systemic PP_i_ has been demonstrated *in vivo* to be sufficient in preventing vascular calcification (Lomashvili et al., 2014). Disease onset of GACI can vary, but the majority of patients present *in utero* through 6 months of age. GACI is associated with a high mortality rate, with death typically occurring from vascular occlusion and additional cardiovascular complications. If left untreated, GACI can result in 85% lethality by 6 months of age (Moran, 1975). A retrospective case study by Rutsch et al. demonstrated that although bisphosphonate treatment reduced the mortality rate during infancy to 35%, 7 of the 17 patients treated with bisphosphonates still died during infancy (Rutsch et al., 2008). The exact cause of death in these individuals was not clear, but because resolution of calcification did not always prevent the subsequent onset of hypertension, untreated arterial vascular stenosis owing to myointimal proliferation instead of calcification could be a contributing factor in these deaths. There are also a few reports of a possible association between long-term use of bisphosphonates and atypical skeletal fractures, but prospective studies will be needed to determine whether there is a causal relationship (Odvina et al., 2005; Otero et al., 2013; Visekruna et al., 2008). In reference to the points mentioned above, a critical and immediate need still exists for an effective treatment for GACI. In this study, we investigated the potential use of ENPP1 enzyme replacement therapy (ERT) for the treatment of GACI.

A previous *in vivo* study performed by Braddock et al. used *Enpp1^asj^* mice (*Asj*), a mouse model with an inactivating missense mutation (p.V246D) in *Enpp1*, to demonstrate that ENPP1 therapy is able to reduce vascular calcification and prolong survival (Albright et al., 2015). Unfortunately, the *Asj* model requires an ‘acceleration diet’ of high phosphate and low magnesium in order to achieve severe vascular calcification. Even with use of an ‘acceleration diet’, aorta mineralization can remain at moderate levels in some mice, with more extensive mineralization found in kidney and other nonvascular sites, increasing the likelihood of these latter sites contributing to the drastic body weight loss and severe lethality (>50% death by 6 weeks age) observed in the model (Li et al., 2013). Similar to *Asj* mice, tip-toe walking (*ttw*) mice, which is another mouse model of GACI with a loss-of-function *Enpp1* mutation, also requires the use of a high-phosphate diet in order to exhibit high levels of vascular mineralization (Huesa et al., 2015; Lomashvili et al., 2014; Watanabe et al., 2017).

In this study, we examined ENPP1 ERT in *Enpp1^asj-2J^* (*Asj-2J*) mice, a model that is more severe than the *Asj* model, possibly due to its ∼40 kb deletion/74 bp insertion in an intron/3′UTR region and a different strain background (Li et al., 2014). The phenotype of *Asj-2J* mice is more translatable to the human disease than that of *Asj* mice, because *Asj-2J* mice develop severe vascular calcification without the use of a nonphysiologically relevant high-phosphate diet and have less mineralization in tissues that are not key to the pathology of GACI (Li et al., 2014). We demonstrate that the *Asj-2J* mice are also advantageous because they exhibit elevated systemic blood pressure, which is believed to be the cause of death in GACI patients. In our investigation, we used *Asj-2J* mice to conduct a more targeted examination of the impact of ENPP1 therapy on vascular calcification and demonstrate, for the first time, a significant improvement in both vascular calcification and cardiovascular function.

## RESULTS

### Natural history of aberrant tissue calcification in *Asj-2J* mice

**Fig. 1.**
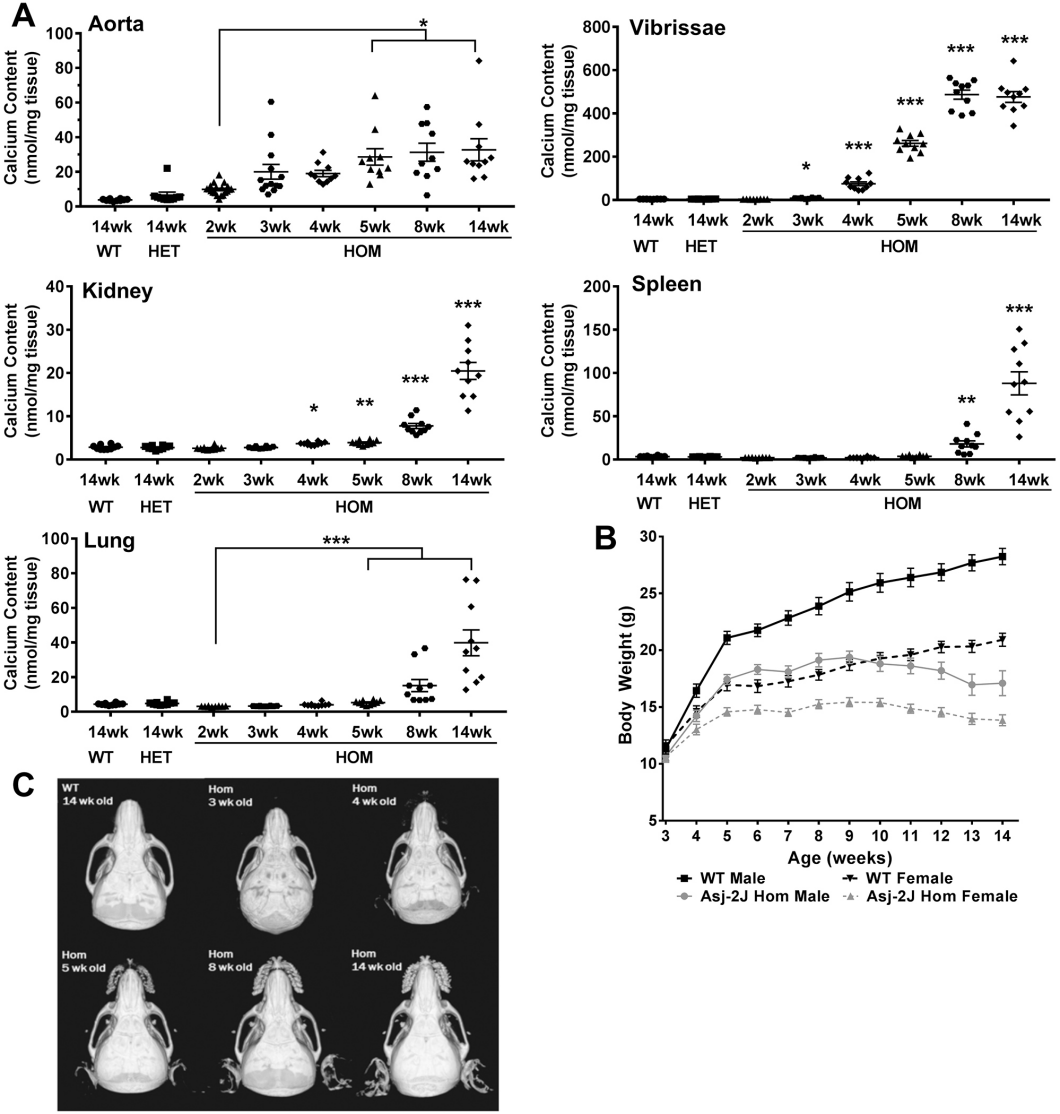
***Asj-2J* mice show a progressive age-dependent increase in calcification in aorta, muzzle skin containing the dermal sheath of the vibrissae, kidney, spleen and lung.** (A) Tissue calcification levels were measured in 2- to 14-week-old WT, *Asj-2J* het (HET) and *Asj-2J* hom (HOM) mice from various tissues using a quantitative calcium assay. Each age group is represented by separate cohorts of mice (*n*=10 mice/group). (B) Body weight was monitored in WT and *Asj-2J* hom male and female mice beginning at 3 weeks of age (*n*=5 mice/group). (C) Representative µCT images of the WT and *Asj-2J* hom mice used in A. Scans focused on the cranial region of mice and show the dermal sheath of vibrissae, ear and ocular areas of mineralization development. All data are mean±s.e.m. *P<0.05, **P<0.005 and ***P<0.0005, compared with 2-week-old *Asj-2J* hom group (by Kruskal–Wallis test, followed by Dunn's multiple comparison test).

A detailed investigation of tissue calcification levels in *Asj-2J* mice was performed in order to better understand the progression of calcification in this model, so that this information could be utilized in a calcification prevention study. Tissue calcification levels were measured using a quantitative calcium assay in aorta, muzzle skin containing the dermal sheath of vibrissae, kidney, spleen and lung from 2- to 14-week-old mice maintained on a chow diet (Fig. 1A). Aortic calcification showed a small, but not statistically significant, increase in 2-week-old *Asj-2J* homozygous (hom) mice in comparison to 5-week-old wild-type (WT) and *Asj-2J* heterozygous (het) mice. Two weeks was the earliest age for which aorta collection was technically feasible. Aortic calcification in *Asj-2J* hom mice continues to progressively increase with age, reaching statistical significance at 5 weeks of age, when there is a 2- to 10-fold elevation. Calcification levels are also increased in a statistically significant manner in *Asj-2J* hom mice compared with WT mice in muzzle skin beginning at 3 weeks of age, kidney at 4 weeks of age, lung at 5 weeks of age and spleen at 8 weeks of age. In terms of severity of calcification, the tissues can be ranked as muzzle skin (vibrissae)>spleen>lung>aorta>kidney, with all tissues reaching maximum levels by 14 weeks of age. Calcification is not detectable in WT and *Asj-2J* het mice up to 14 weeks of age in any of the tissues examined. By 14 weeks of age, *Asj-2J* hom mice show observable changes such as reduced mobility and a gaited walk. They also begin to demonstrate reduced viability, signs of which are reflected in body weight loss (Fig. 1B). Body weight monitoring beginning at 3 weeks of age demonstrates that *Asj-2J* hom mice have reduced body weight gain compared with WT mice and, by ∼9-10 weeks of age, this transitions to body weight loss, with males and females showing an average of 11% and 13% body weight loss, respectively.

Mineralization of the vasculature of the heart, including the aortic regions, was also examined histologically by Alizarin Red staining, and severity was graded on a 0-4 scoring scale based on the number of mineralized foci that were present, the relative size of the foci that were present and the staining intensity. Representative images for mineralization scores of 2, 3 and 4 are shown in Fig. S1A-C. Vascular mineralization in *Asj-2J* mice appeared to present first in the tunica intima regions of vessels and began extending into the tunica media region as the disease progressively advanced with age. Alizarin Red staining showed a similar progression of mineralization compared with the quantitative calcium assay (Fig. 1A; Fig. S1D).

Tissue calcification data from *Asj-2J* mice were used to establish a therapeutic treatment window for a calcification prevention study, which would begin at 2 weeks of age when calcification is very low/absent in all tissues, and continue through 5 weeks of age, when calcification levels reach a maximum of an average 2.5-fold and 70-fold increase in aorta and muzzle skin, respectively. Although calcification of muzzle skin is not relevant to humans, it was measured as an additional method to verify therapeutic efficacy and has the advantage that it can also be visualized by micro computed tomography (µCT) imaging. μCT imaging shows a similar calcification level in the muzzle skin region compared with that measured with the quantitative calcium assay. At 5 weeks of age, there is not yet a significant increase in kidney, spleen and lung calcification in *Asj-2J* hom mice, which is preferable because the aorta is the tissue most clinically relevant to the disease pathology of GACI patients.

### Pharmacokinetic/pharmacodynamic study of rhENPP1 protein therapeutic efficacy in *Asj-2J* mice

**Fig. 2.**
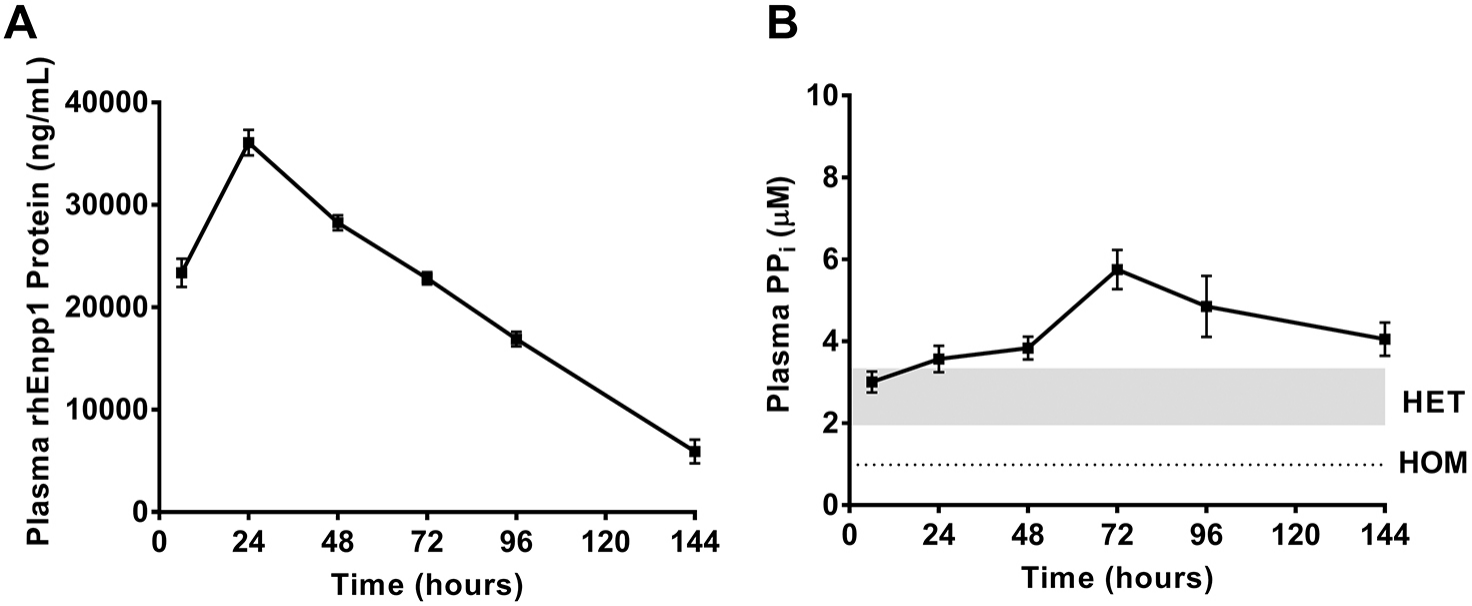
**PK/PD analysis of rhENPP1 demonstrates elevated rhENPP1 protein and plasma PP_i_ for at least 144 h in *Asj-2J* mice.** Five-week-old *Asj-2J* hom mice were subcutaneously injected with 5000 U/kg rhENPP1 or vehicle, and plasma was collected at 6, 24, 48, 72, 96 or 144 h postinjection. (A) Plasma rhENPP1 protein levels are increased in *Asj-2J* hom mice by 6 h post-rhENPP1 injection and remain elevated for at least 144 h. C_max_ is reached at ∼24 h postinjection. rhENPP1-treated *Asj-2J* hom mice are compared with WT, *Asj-2J* het or vehicle-treated *Asj-2J* hom mice, which all measure below the LLOQ for the assay (<50 ng/ml). (B) Plasma PP_i_ levels are elevated by 6 h post-rhENPP1 injection and remain at or above *Asj-2J* het PP_i_ levels for at least 144 h. C_max_ is reached at ∼72 h postinjection. rhENPP1-treated *Asj-2J* hom mice are compared with WT, *Asj-2J* het or vehicle-treated *Asj-2J* hom mice. Gray shading represents plasma PP_i_ levels in *Asj-2J* het mice and the dotted line represents levels in *Asj-2J* hom mice. Each timepoint in A and B is represented by a separate cohort of mice with *n*=8 mice/group, except the WT and vehicle-treated *Asj-2J* hom groups which have *n*=6 mice/group and *n*=3 mice/group, respectively. All data are mean±s.e.m.

A single-dose pharmacokinetic/pharmacodynamic (PK/PD) study with rhENPP1 was performed in order to determine the dosing regimen required for a multidose chronic study (Fig. 2). rhENPP1 dosage was based on ENPP1 activity units instead of protein concentration in order to reduce interstudy variation owing to usage of different lots of rhENPP1 protein. Therefore, in this study, *Asj-2J* hom mice were injected with rhENPP1 at a 5000 U/kg dose (10.1 mg/kg, lot#1). In response to a single subcutaneous injection of 5000 U/kg rhENPP1, plasma rhENPP1 protein was elevated by 6 h in *Asj-2J* mice, reaching its maximum concentration observed (C_max_) at ∼24 h, and remained elevated until 144 h postinjection (Fig. 2A). rhENPP1 levels in WT, *Asj-2J* het and vehicle-treated *Asj-2J* hom mice were all below the lower limit of quantitation (LLOQ) for this assay. Our previous studies have shown that plasma rhENPP1 activity has a similar profile to plasma rhENPP1 protein levels (data not shown). The pharmacodynamic effect of rhENPP1 was monitored by measuring the production of PP_i_, which results from ENPP1-mediated ATP hydrolysis. Similar to the plasma rhENPP1 protein profile, plasma PP_i_ levels were also elevated in *Asj-2J* hom mice by 6 h after rhENPP1 injection, in comparison to levels in vehicle-treated *Asj-2J* hom mice, reaching levels at or above *Asj-2J* het and WT PP_i_ levels (het, 1.9-3.3 µM; WT, 2.1-4.1 µM, respectively) (Fig. 2B). Similar to previous reports of plasma PP_i_ levels in *Asj-2J* hom mice, plasma PP_i_ in vehicle-treated *Asj-2J* hom mice is very low at <0.94 µM. A minimum threshold of *Asj-2J* het plasma PP_i_ levels is believed to be sufficient for disease prevention because *Asj-2J* het mice do not show an increase in tissue calcification (Fig. 1A) or any other observable signs of disease (Li et al., 2014). In contrast to the rhENPP1 protein profile of *Asj-2J* mice in this PK/PD study, the PP_i_ profile was shifted to the right, reaching its C_max_ only at 72 h (Fig. 2B). Plasma PP_i_ remained elevated above WT levels for at least 144 h after a single injection of rhENPP1, suggesting that a weekly dosing regimen would be sufficient to maintain normal PP_i_ in *Asj-2J* mice.

### rhENPP1 treatment prevents calcification development in *Asj-2J* mice

**Fig. 3.**
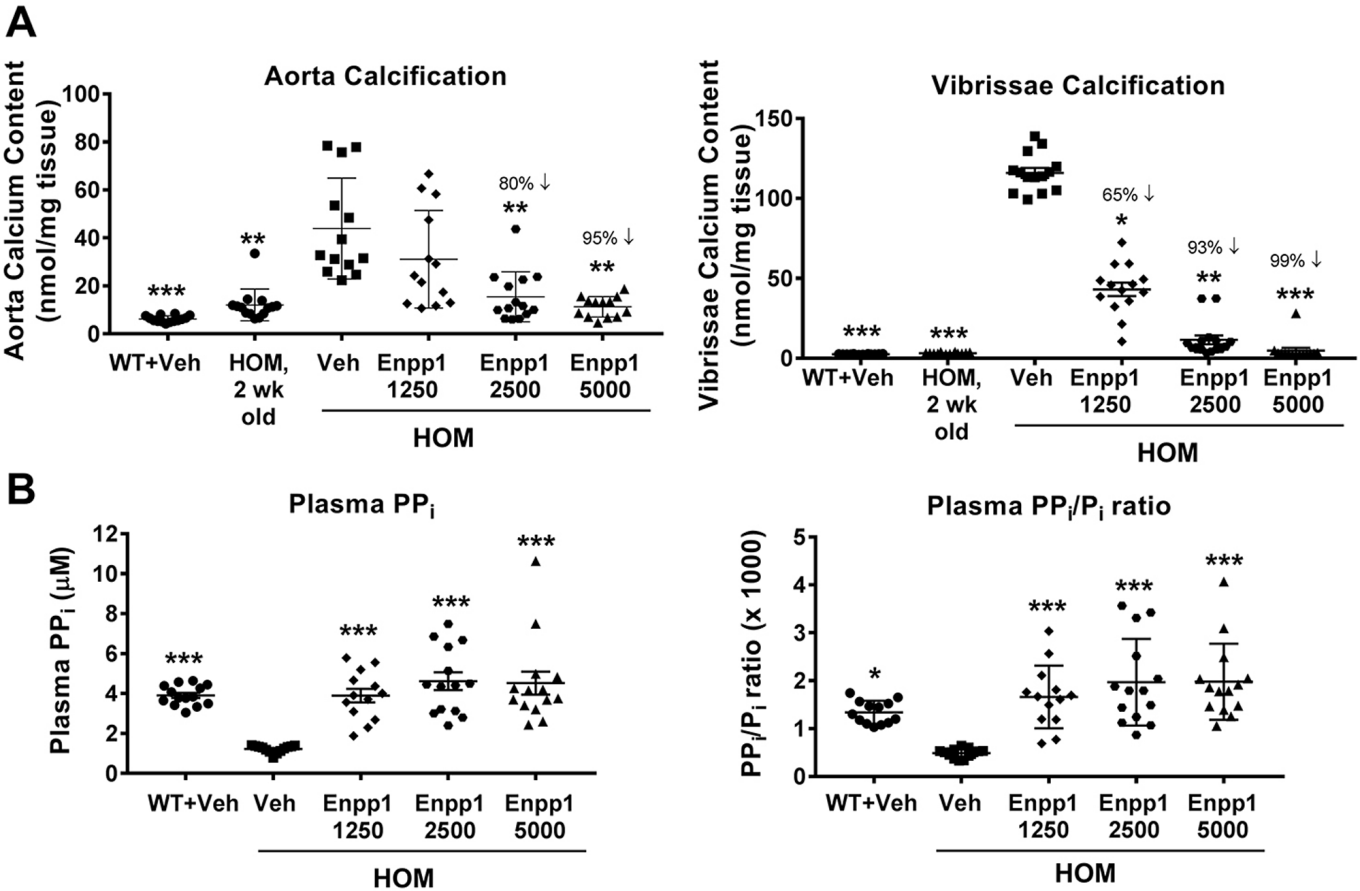
**rhENPP1 treatment for 3 weeks completely prevents calcification development in the aorta and muzzle skin containing the dermal sheath of vibrissae in *Asj-2J* mice.** In order to assess ENPP1 effect on calcification prevention, mice were dosed beginning at 2 weeks of age (when *Asj-2J* mice do not yet show significantly elevated calcification) with 1250, 2500 or 5000 U/kg rhENPP1 (lot#1) or vehicle (Veh) weekly by subcutaneous injection for a period of 3 weeks. All mice also received an injection of anti-CD4 mAb 1 day prior to each rhENPP1 dose (see Materials and Methods for additional details). Each treatment group consists of a cohort of *n*=14 mice/group. Tissues and plasma were collected 7 days after the final dose. (A) Calcification levels in aorta and muzzle skin containing the dermal sheath of vibrissae were measured using a quantitative calcium assay. Two-week-old *Asj-2J* hom mice were used as an estimated baseline measurement of calcification levels before initiation of the 3-week treatment. (B) Plasma PP_i_ and calculated PP_i_/P_i_ ratio were measured in mice. All data are mean±s.e.m. All statistical analyses were performed relative to vehicle-treated *Asj-2J* hom mice. *P<0.05, **P<0.005 and ***P<0.0005 (by Kruskal–Wallis test, followed by Dunn's multiple comparison test).

In order to determine the efficacy of rhENPP1 therapy in calcification prevention, *Asj-2J* hom mice were treated with rhENPP1 beginning at 2 weeks of age, an age that precedes the development of significant tissue calcification. Mice were treated with vehicle or rhENPP1 at 1250, 2500 or 5000 U/kg (lot #1; 2.5, 5 or 10 mg/kg, respectively) doses by weekly subcutaneous injection for a period of 3 weeks (Fig. 3). In previous studies we observed anti-drug antibody (ADA) development and the subsequent loss of detectable levels of rhENPP1 protein in *Asj-2J* mice after 3 doses of rhENPP1 within a 15-day period (Fig. S2A,B). This is most likely the result of injecting mice with an ENPP1 protein that is recombinant and from a different species. Numerous rodent studies have shown that co-administration of anti-CD4 monoclonal antibody (mAb) can prevent ADA development in response to a protein replacement therapy and therefore this strategy was utilized here (Rice and Bucy, 1995; Sun et al., 2014). Flow cytometry analysis confirmed >90% depletion of CD4^+^ T cells in peripheral blood from *Asj-2J* mice 7 days after a single 300 µg injection with an anti-CD4 mAb (Fig. S3A). Anti-CD4 mAb treatment did not affect body weight or calcification levels in the aorta and muzzle skin (Fig. S3B).

Therefore, in order to prevent ADA development and maximize our efficacy in the *Asj-2J* model, animals were treated with anti-CD4 mAb 1 day prior to each dose of rhENPP1 or vehicle (Fig. 3). By the end of the 3-week study, vehicle-treated *Asj-2J* mice showed an average 4.4-fold increase in aorta calcification and a 36-fold increase in calcification of muzzle skin containing the dermal sheath of vibrissae compared with baseline levels in 2-week-old *Asj-2J* mice (Fig. 3A). Treatment of *Asj-2J* mice with rhENPP1 caused a dose-dependent reduction in both aorta and muzzle skin calcification, showing >95% calcification prevention at the 5000 U/kg dose. Both tissues also showed significant calcification reduction at the 2500 U/kg dose. Plasma PP_i_ and inorganic phosphate (P_i_) levels were measured 7 days after the final ENPP1 dose, and showed that PP_i_ and the PP_i_/P_i_ ratio were increased to WT levels with all 3 rhENPP1 doses, with no significant change in P_i_ levels (Fig. 3B).

### rhENPP1 treatment improves systemic blood pressure and cardiac function in *Asj-2J* mice

**Fig. 4.**
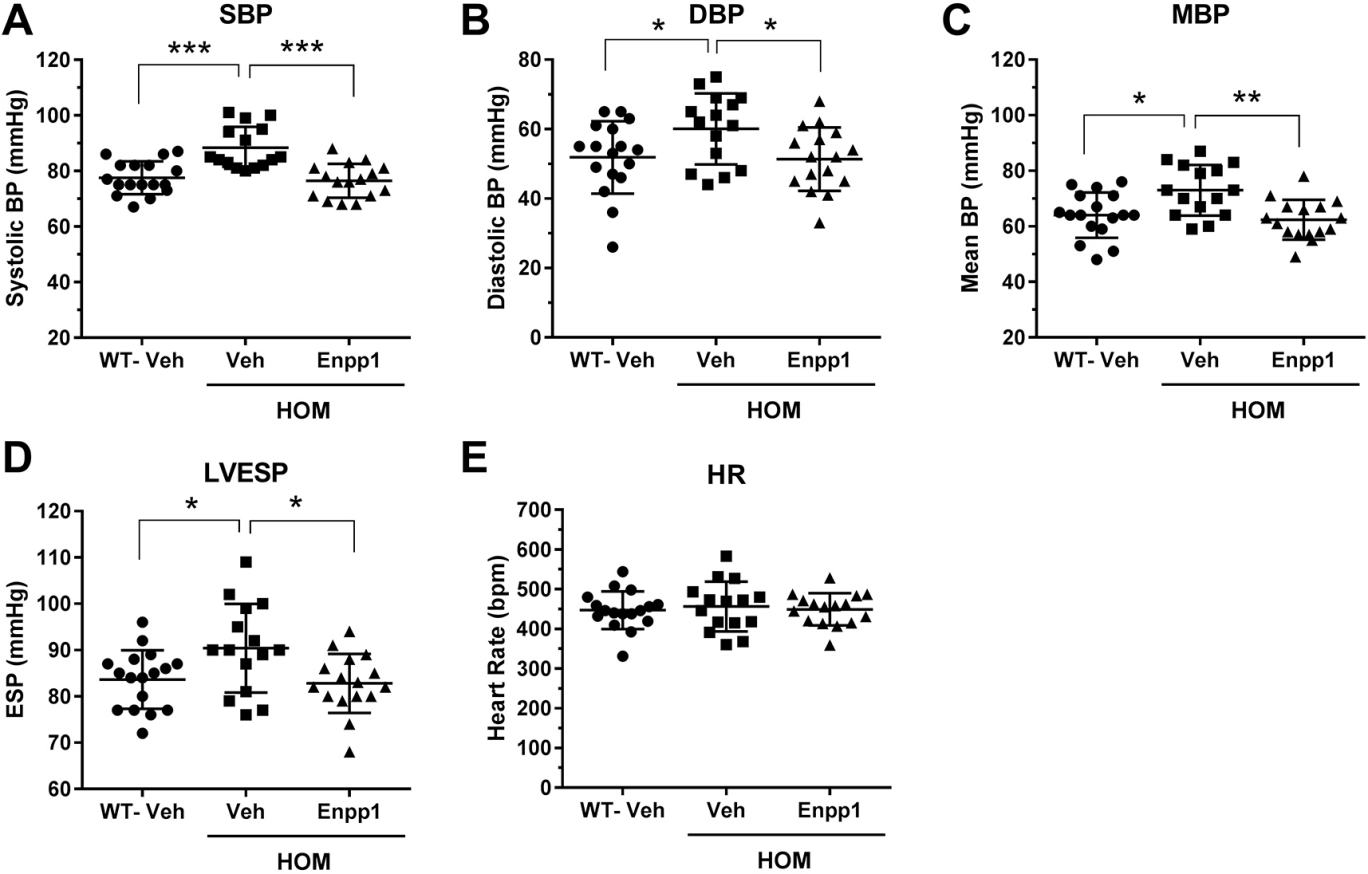
**rhENPP1 treatment for 6 weeks reduces systemic blood pressure and left ventricular pressure in *Asj-2J* mice.** Mice were dosed beginning at 2 weeks of age with 2100 U/kg rhENPP1 (lot#2) or vehicle every other day by subcutaneous injection for a period of 6 weeks. (A-E) Terminal hemodynamics were performed on isoflurane-anesthetized mice (*n*=15 mice/group) (as described in the Materials and Methods) to assess effects on systolic blood pressure (SBP) (A), diastolic blood pressure (DBP) (B), mean blood pressure (MBP) (C), left ventricular end-systolic pressure (LVESP) (D) and heart rate (HR) (E). Summary data (shown as raw values) are shown and all data are mean±s.e.m. *P<0.05, **P<0.005 and ***P<0.0005 (by one-way ANOVA, followed by Tukey's multiple comparison test).

Further characterization of *Asj-2J* mice by terminal hemodynamics and echocardiography (ECHO) imaging were performed in order to determine whether the model is sufficient to study the hypertension and cardiac changes evident in GACI patients. Owing to technical limitations of performing the terminal hemodynamic procedure in young mice because of their small body size, 2-week-old mice were treated with rhENPP1 for a period of 6 weeks and the procedure was performed in 8-week-old mice. Initial characterization of 8-week-old *Asj-2J* mice by terminal hemodynamics revealed that they had elevated systemic blood pressure, with increased systolic, diastolic and mean blood pressure, as well as elevated left ventricular end-systolic pressure (LVESP) versus aged-matched WT controls (Fig. 4A-D). *Asj-2J* mice had comparable heart rates to those of WT control mice (Fig. 4E).

**Fig. 5.**
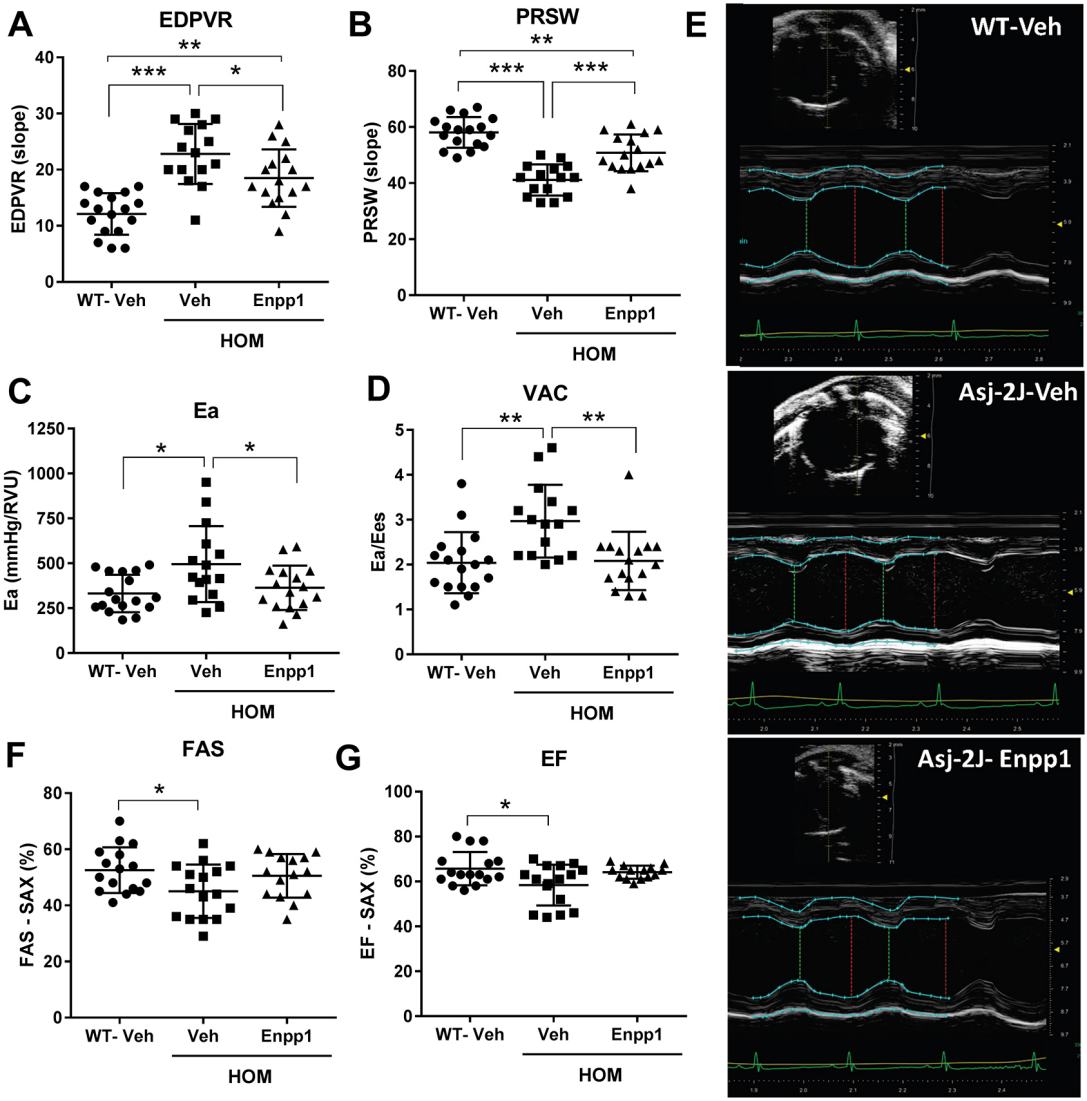
**rhENPP1 treatment for 6 weeks improves cardiac function in *Asj-2J* mice.** Mice were dosed beginning at 2 weeks of age with 2100 U/kg rhENPP1 (lot#2) or vehicle every other day by subcutaneous injection for a period of 6 weeks. Pressure volume loops were performed on isoflurane-anesthetized mice to assess the impact of rhENPP1 treatment on different parameters of cardiac function. (A,B) Summary data (shown as raw values) of the end-diastolic pressure volume relationship (EDPVR) (A) and preload recruitable stroke work (PRSW) (B) were measured as indicators of effects on myocardial compliance and contractility, respectively. (C,D) Arterial elastance (Ea) (C) and ventriculo-arterial coupling (VAC) (D) were measured as a reflection of changes in heart workload and mechanical efficiency, respectively. (E) ECHO imaging was performed on isoflurane-anesthetized mice using 1-dimensional M-mode view. Representative short-axis M-mode ECHO images taken at the mid-papillary level of the left ventricle are shown for each treatment group. (F,G) The lumen area of the left ventricle in the ECHO images were used to evaluate left ventricular fractional area shortening (FAS) (F) and ejection fraction (EF) (G). All data are mean±s.e.m. Each treatment group consisted of a cohort of *n*=15 mice/group. *P<0.05, **P<0.005 and ***P<0.0005 (by one-way ANOVA, followed by Tukey's multiple comparison test).

To assess the consequence of the hypertension on cardiac function, left ventricular function was evaluated by performing pressure volume loops. The linear slope of the end-diastolic pressure volume relationship (EDPVR) was significantly increased and the preload recruitable stroke work (PRSW) was significantly decreased, indicating reduced compliance and contractility, respectively, in the disease model (Fig. 5A,B). Consistent with elevated arterial mineralization, *Asj-2J* mice had increased arterial elastance (Ea) and ventriculo-arterial coupling (VAC), suggesting increased workload of the heart and reduced mechanical efficiency, respectively (Fig. 5C,D). ECHO imaging revealed similar myocardial dysfunction in *Asj-2J* mice, with significantly reduced fractional area shortening (FAS) and ejection fraction (EF) compared with WT mice (Fig. 5E-G).

To test whether rhENPP1 ERT is effective at preventing the observed hypertension and cardiac changes, a treatment study with rhENPP1 was performed. A different lot of rhENPP1 protein was utilized in this study, having a lower bioactivity and reduced exposure than the lot used in the calcification prevention study, owing to different conditions used during the protein production process. As a result, mice required more frequent dosing with this lot of rhENPP1 protein and were, therefore, injected with rhENPP1 subcutaneously every other day at a 2100 U/kg (lot#2; 4 mg/kg) dose. An anti-CD4 treatment was not incorporated in this study, thus putting the mice in this study at risk for ADA development. In this setting, rhENPP1 treatment of *Asj-2J* mice for 6 weeks significantly reduced systolic blood pressure [−13±2% (mean±s.e.m.)], diastolic blood pressure [−14±4% (mean±s.e.m.)], mean blood pressure [−15±2% (mean±s.e.m.)] and LVESP [−8±2% (mean±s.e.m.)] versus the vehicle group, and the values were normalized to WT levels (Fig. 4A-D).

Myocardial compliance [EDVPR, −19±6% (mean±s.e.m.)] and contractility [PRSW, 24±4% (mean±s.e.m.)] were significantly improved in the *Asj-2J* mice, but remained statistically different from those in WT mice (Fig. 5A,B). Treatment also significantly reduced the workload of the heart [Ea, −26±6% (mean±s.e.m.)] in these mice and increased its mechanical efficiency [VAC, −30±6% (mean±s.e.m.)] to WT values (Fig. 5C,D). ECHO image analysis showed that although rhENPP1-treated *Asj-2J* mice did not demonstrate significantly improved FAS and EF compared with vehicle-treated *Asj-2J* mice, these parameters were increased to levels that were not significantly different from those of WT mice (Fig. 5E-G). Altogether, the cardiovascular effects suggest that rhENPP1 treatment was able to improve hypertension and some aspects of cardiac function in *Asj-2J* mice. However, the absence of anti-CD4 mAb co-administration with rhENPP1 treatment resulted in the development of ADAs, undetectable rhENPP1 protein, and no improvements in plasma PP_i_ and aorta calcification levels during the 6-week rhENPP1 treatment course (Fig. S4A,B).

## DISCUSSION

In this study, we demonstrate, for the first time, that ENPP1 replacement therapy can significantly reduce both arterial calcification and hypertension in a mouse model of GACI. To date, many therapeutics for GACI have been investigated in the *Asj* model, which requires a nonphysiological ‘acceleration diet’ in order to trigger and enhance the GACI disease pathology of increased arterial mineralization (Albright et al., 2015; Li et al., 2013, 2016). Owing to the reduction in body weight caused by the ‘acceleration diet’, the *Asj* model is not ideal for studying the implications of ENPP1 therapy on blood pressure and cardiac function. Many other ENPP1-deficient models also require being challenged with an ‘acceleration diet’ in order to achieve levels of aorta calcification similar to 5-week-old Asj-2J mice (Cecil and Terkeltaub, 2011; Huesa et al., 2015; Watanabe et al., 2017). For these reasons, we performed our studies in *Asj-2J* mice, a model that we found to more accurately recapitulate clinical symptoms of GACI. These mice present with severe arterial calcification with minor secondary calcification in nonvascular tissues (Fig. 1A). Therefore, this model enabled us to perform a more accurate assessment of the effect of ENPP1 therapy on the hypertension that is a key pathological feature of GACI and ultimately leads to patient death.

Treatment of *Asj-2J* mice with rhENPP1 resulted in almost complete prevention of aorta calcification development (Fig. 3A), and significantly reduced systemic blood pressure and left ventricular pressure (Fig. 4A-D). rhENPP1-mediated effects on hypertension also successfully translated into additional improvements in myocardial compliance, contractility, heart workload and global cardiovascular efficiency (Fig. 5A-D). In the 6-week rhENPP1 treatment study, ADAs developed against rhENPP1 because mice were injected with a human recombinant protein without co-administration of anti-CD4 mAb to prevent ADA development (Fig. S4B). However, because ADAs in our studies take ∼2-3 weeks to develop (Fig. S2), rhENPP1 was still able to mediate its effects on the disease during this early treatment window preceding ADA development. We have previously observed ∼50% reduction in aorta calcification in *Asj-2J* mice in the initial 3 weeks of treatment, despite a moderate level of ADAs (data not shown).

However, at the end of our 6-week treatment of *Asj-2J* mice, rhENPP1 did not demonstrate an effect on plasma PP_i_ or aorta calcification owing to higher ADA levels (Fig. S4A,B). Although there have been reports that transient increases in plasma PP_i_ can be sufficient to inhibit calcification in Abcc6^−/−^ and *ttw* models, there was no effect on the endpoint of aorta calcification in our study (Dedinszki et al., 2017). The observed improvements in blood pressure and cardiac function in *Asj-2J* mice at the end of the 6-week treatment course, despite a lack of effect on aorta calcification, suggest that there could have been a critical window for efficacy early in disease development, during which rhENPP1 could have impacted a noncalcification/non-PP_i_ mediated mechanism. The impact on this mechanism could be what is responsible for the sustained improvements in cardiovascular function.

In addition to PP_i_ production, ENPP1 hydrolysis of ATP can also produce AMP, which subsequently leads to the production of adenosine via ecto-5′-nucleotidase (CD73; also known as Nt5e). Although adenosine levels were not measured in this study, adenosine has been reported in previous literature to be a potent vasodilator, and to inhibit VSMC proliferation, stimulate cardiac contractility and have cardioprotective effects during ischemia/reperfusion injury (Chandrasekera et al., 2010; Dubey et al., 1996, 2000; Jordan et al., 1997). These adenosine effects appear to be mainly mediated through signaling via the adenosine A_2A_ receptor (A_2A_R; also known as Adora2a), which is the adenosine receptor most highly expressed by vascular smooth muscle cells and the endothelium (Lewis et al., 1994). Further studies will be needed to determine whether rhENPP1 affects adenosine levels and myointimal proliferation, and to understand whether they play a role in the improvements in hypertension and cardiac function in rhENPP1-treated *Asj-2J* mice.

In our studies, *Asj-2J* mice, which are in the BALB/cJ strain background, were found to be more susceptible to ADA development in response to rhENPP1 than other mouse strains (data not shown). ADAs were not observed in *Asj* mice in a C57Bl/6J strain that received a similar rhENPP1 treatment course. An enhanced ADA response in BALB/cJ strain mice was also reported by Shomali et al. (2015). This susceptibility is speculated to be due to the Th2-dominant response that occurs in BALB/cJ mice, as opposed to the Th1-dominant response that occurs in C57Bl/6J mice (Watanabe et al., 2004).

In conclusion, our studies demonstrate that rhENPP1 ERT is an effective treatment for GACI by treating 2 attributes of the disease. rhENPP1 therapy successfully prevents calcification development and we show, for the first time, that it can also have a major impact on hypertension and cardiovascular function in a GACI disease model. Because hypertension is the leading cause of cardiac failure in GACI patients, rhENPP1 ERT could be important in resolving the disease more effectively and in a greater number of patients than bisphosphonates (Rutsch et al., 2008; Thiaville et al., 1994). Further studies will be necessary to examine the therapeutic window for rhENPP1 ERT in GACI patients, and whether it has capabilities beyond being used as a disease prevention tool. Calcification reversal will likely be more difficult to achieve owing to the pronounced stability and insolubility of hydroxyapatite, but chelating agents and vitamin K supplementation have been shown to reduce existing calcification in rat arterial calcification models. The role of rhENPP1 in calcification regression will need to be evaluated in the future, but will likely depend on the extent of the calcification present and require long-term treatment (Lei et al., 2013; Schurgers et al., 2007). rhENPP1 ERT could also be a promising therapy for other diseases caused by loss-of-function mutations in *ENPP1* or low plasma PP_i_ levels, or characterized by ectopic vascular calcification, such as pseudoxanthoma elasticum, autosomal-recessive hypophosphatemic rickets and chronic kidney disease. Further investigation of rhENPP1 therapy is warranted in these diseases, but suggests the possibility of a potential cluster of rhENPP1-treatable vascular calcification disorders.

## MATERIALS AND METHODS

### Animals and treatment studies

BALB/cJ-*Enpp1^asj-2J^*/GrsrJ mice were obtained from The Jackson Laboratory (strain #019107). Mice were maintained on a 12-h light and dark cycle with *ad libitum* access to water and standard chow diet (5058 or 5008; LabDiet, St Louis, MO, USA). Breeding pairs of *Asj-2J* het mice were fed a 5058 diet, and their litters – comprising the experimental cohort of mice (referred to hereafter as WT, *Asj-2J* het, and *Asj-2J* hom or *Asj-2J*) – were transferred to a 5008 diet at weaning age. All animal studies were conducted at Alexion Pharmaceuticals in Lexington, MA, USA, in a pathogen-free animal facility using protocols approved by the Institutional Animal Care and Use Committee. In all treatment studies, mice were treated with rhENPP1 or vehicle subcutaneously beginning at 2 weeks of age at the indicated doses and dose frequency. All studies were performed on groups made up of 50% males and 50% females, unless otherwise noted. Animal dosage of rhENPP1 was based on enzymatic activity per kilogram body weight (U/kg) instead of milligram per kilogram body weight (mg/kg) in order to increase consistency between different lots of protein. Animal studies utilized 2 different lots of rhENPP1. Lot #1 of rhENPP1 was ∼490 U/mg activity and 1.58 mg/ml, and was utilized in the PK/PD and calcification prevention study. Lot #2 of rhENPP1 was 530 U/mg activity and 1.61 mg/ml, and was utilized in the cardiac function study. Some animals were also co-administered with an anti-mouse purified functional grade CD4 mAb, clone GK1.5 from Affymetrix eBioscience (San Diego, CA, USA) to prevent development of ADAs against rhENPP1. Anti-CD4 treatment was given intraperitoneally 1 day prior to each dose of rhENPP1 and consisted of 500 µg for the first dose, followed by 200 µg for each subsequent dose. Only animals in the calcification prevention study received this anti-CD4 mAb treatment course.

### μCT imaging

Mice were scanned using the Quantum GX µCT (Perkin Elmer, Hopkinton, MA, USA) with a focus on the cranial region of the mice (36 FOV, 2 min scans, 72 µm voxel size resolution). Images were processed using Quantum software v 3.0.13.1100.

### rhENPP1 construct design and protein production

The rhENPP1 construct was designed so that the expressed protein had the soluble portion of human ENPP1 (NCBI accession NP_006199) fused to the human IgG1 Fc domain at the C-terminus, as previously described (Albright et al., 2015). The fusion protein was expressed and produced from a stable Chinese hamster ovary cell line using a modern fed-batch production bioreactor process that was animal component-free and fully chemically defined, intended to enhance total sialic acid content of the rhENPP1 fusion protein. rhENPP1 was purified using a GE MabSelect SuRe affinity Protein A column (GE Healthcare Life Sciences, Marlborough, MA, USA), followed by anion exchange polishing using a salt gradient elution. This material was then buffer exchanged using ultrafiltration/diafiltration into a 20 mM HEPES, 140 mM sodium chloride, pH 7.3 buffer. This HEPES/NaCl buffer also served as the vehicle control in all animal studies. All rhENPP1 protein used in animal studies was tested for endotoxin using Endosafe cartridge technology from Charles River Labs (Wilmington, MA, USA) and was determined to be <0.3 EU/mg.

### Terminal hemodynamic measurements and ECHO measurements

All hemodynamic and ECHO measurements were performed by QTest Labs (Columbus, OH, USA). Each mouse was anesthetized with ∼3% isoflurane positioned in dorsal recumbence. Anesthesia was maintained using 1.5% isoflurane with 100% oxygen via a nose cone. Mice were positioned right lateral recumbent, and transthoracic M-mode examinations were assessed at the mid-papillary region to evaluate left ventricular FAS and EF via ECHO (Visual Sonics - VEVO 2100). Following the conclusion of the ECHO, a cut down was performed to expose the trachea and carotid artery. Via a tracheotomy, a 22 G angiocatheter was used to endotracheally intubate and ventilate with an adjustable small animal ventilator (Harvard Apparatus, Holliston, MA, USA) (∼150 breaths/min, ∼0.5 ml tidal volume with 100% oxygen). A 1.2F high-fidelity conductance micromanometer catheter (SciSense; Transonic Systems, Ithaca, NY, USA) was inserted into the right carotid artery to measure arterial pressures. After a baseline reading for ∼10-15 min, the catheter was advanced retrograde across the aortic valve and into the LV chamber, by way of the right carotid artery, in order to simultaneously determine left ventricular pressure and volume (via conductivity of ventricular blood). To generate a family of pressure-volume curves/loops, venous return was temporarily reduced by using forceps to gently press on the inferior vena cava in the abdomen, below the diaphragm, or by occluding the ventilator's exhaust line for several breaths (Valsalva maneuver) in order to increase intrathoracic pressure. The resulting left ventricular pressure and volume data were analyzed offline (IOX/ECG Auto; EMKA Technologies, Paris, France) in order to generate relationships representing the contractile state of the myocardium.

### rhENPP1 activity assay

An enzymatic endpoint assay for ENPP1 activity was developed based its ability to hydrolyze thymidine 5′-monophosphate p-nitrophenyl ester sodium salt (T4510; Sigma-Aldrich, Allentown, PA, USA). A prior lot of rhENPP1 served as the stock ENPP1 used for generating all standard curves in this assay. Test samples and standards were diluted in assay buffer (0.11 M Tris pH 9.0, 0.11 M NaCl, 15 mM MgCl_2_). All standards and samples (10 µl) were incubated with 1 mM substrate (90 µl) and incubated in the dark for 30 min at 37°C. Enzymatic activity was determined spectrophotometrically at 450 nm using SpectraMax M5 (Molecular Devices, Sunnyvale, CA, USA), with 1 unit of enzyme defined as 1 µmol/min.

### rhENPP1 protein assay

Human ENPP1 was detected by Meso QuickPlex SQ 120 (MSD, Rockville, MD, USA) using 2 polyclonal rabbit antibodies generated in-house against human ENPP1. MSD standard plates were coated with 1 µg/ml ENPP1 coating antibody overnight at 4°C and then washed 3× with 1× PBS, 0.3% Tween-20 buffer. The plates were blocked with 1% bovine serum albumin (BSA) in PBS for 1 h at room temperature (RT) and then washed as previously described. Standards and samples diluted in dilution buffer containing 5% of the appropriate matrix were then loaded onto plates, incubated for 1 h at RT, and then washed as described. The reference standard used was the specific lot of rhENPP1 being analyzed. Sulfo-tagged ENPP1 detection antibody was prepared using an MSD Gold sulfo-tag NHS-Ester and was added to each well at 1 µg/ml, incubated at RT for 1 h, and then washed as described. In the final step, plates were incubated with MSD Read buffer (R92TC-2) for detection of bound sulfo-tag antibodies. Plates were read using the MSD system and all data were extrapolated using the software provided with the MSD system.

### rhENPP1 anti-drug antibody assay

Anti-drug antibodies for rhENPP1 were detected by the MSD method. Biotinylated and sulfo-tagged rhENPP1 were prepared using standard methods using an EZ-Link NHS-PEG4-Biotinylation kit (Thermo Fisher Scientific, Waltham, MA, USA) and an MSD Gold sulfo-tag NHS-Ester, respectively. The standard curve was prepared using an in-house rabbit anti-human polyclonal ENPP1 antibody diluted 1:20 in pooled mouse serum. Sample or standard (25 µl) was added to each well of a 96-well polypropylene plate, followed by the addition of 50 µl of a 0.5 µg/ml mixture of biotinylated and sulfo-tagged rhENPP1 (1:1 ratio) and incubated for 1 h at RT. Separately, 150 µl of a 3% BSA/PBS blocking buffer was added to each well of a 96-well Streptavidin Gold plate (MSD) and incubated for 1 h. Each well was washed 1× with wash buffer, and then 50 µl of the incubated mixture of sample/control, biotinylated rhENPP1 and sulfo-tagged rhENPP1 was added. The plate was incubated for 1 h and washed 3 times. Finally, 150 µl of 2× MSD Read buffer (R92TC-2) was added and the plate was read on the MSD instrument. The cutoff point for potential ADA-positive samples was based on the average values of pre-bleeds or vehicle-treated mouse samples.

### Quantitative tissue calcium assay

Tissues were decalcified in 4 µl 1 N HCl/mg tissue overnight. For aorta, the aortic arch and the descended aorta region was used in the analysis. The extract was tested in a colorimetric assay utilizing the o-cresolphthalein complexone method (Lomashvili et al., 2014). The standard curve utilized a calcium reagent prepared in 1 N HCl. Standards and samples (5 µl) were mixed with working reagent (250 µl), for a final concentration of 270 mM 2-amino-2-methyl-1-propanol, 5.2 mM 8-hydroxyquinolone and 100 µM o-cresolphthalein. The reaction was incubated for 5 min and absorbance read at 575 nm using a SpectraMax M5. Tissue calcium content was normalized to tissue weight. All reagents used were obtained from Sigma-Aldrich.

### PP_i_ and P_i_ assays

Platelet-depleted plasma was used for PP_i_ and P_i_ analysis. Briefly, an Amicon Ultra-2 Centrifugal Filter unit with Ultracel-30 membrane from EMD Millipore (Billerica, MA, USA) was pre-wet by centrifuging hydroxyapatite-treated water at 14,000 ***g***. This was followed by centrifugation of freshly collected heparinized plasma through the filter at 14,000 ***g*** for 30 min. Filtered plasma was immediately flash-frozen and stored at −80°C prior to PP_i_ and P_i_ analysis. Plasma PP_i_ was measured using PPiLight™ Inorganic pyrophosphate assay (Lonza, Allendale, NJ, USA) with minor modifications. Filtered mouse plasma was diluted 4-fold using hydroxyapatite-treated water prior to analysis. For each replicate, 32.6 µl of diluted plasma is combined with 25 µl of assay reagent in triplicate wells in 384-well format, mixed with gentle tapping and incubated for 1 h at 25°C in the dark. Luminescence was measured using a Flexstation M3 plate reader (Molecular Devices). Owing to observed matrix effect of plasma in this assay, PP_i_-depleted mouse plasma was prepared for use as background matrix for preparation of the PP_i_ calibrator curve. Briefly, pooled mouse plasma was treated with 0.5 U/µl inorganic pyrophosphatase at 37°C for 1 h, heat-inactivated at 70°C for 1 h and filtered through the Amicon filter. Flow-through filtrate was pooled, aliquoted and stored at −80°C. PP_i_-depleted mouse plasma was similarly diluted 4-fold using hydroxyapatite-treated water prior to use. Plasma P_i_ was analyzed using a colorimetric molybdate-based assay on the AU480 clinical analyzer (Beckman Coulter, Danvers, MA, USA).

### Detection of CD4^+^ T cells by flow cytometry

Whole blood from mice was tested for the presence of CD4^+^ T cells as follows: 100 µl whole blood in EDTA was incubated with APC-conjugated anti-mouse CD4 antibodies (116013, clone RM4-4; BioLegend, San Diego, CA, USA; 5 µl/sample, 1:2 dilution) for 30 min at ambient temperature. Next, 2 ml/sample Mouse Red Blood Cell Lysis Buffer (IBB-198; Boston Bioproducts, Ashland, MA, USA) was added and samples incubated for 10 min at ambient temperature in the dark, followed by 2 washes in 1 ml/sample FACS Buffer (1% BSA in dPBS, 0.1% sodium azide). Cells were resuspended in 250 µl FACS Buffer and accessed by flow cytometry (20,000 events/sample collected) using a DxP6 flow cytometer (Cytek Development, Freemont, CA, USA). In a follow-up trial, staining was performed as described above with the addition, prior to acquisition by flow cytometry, of propidium iodide staining solution (556463; BD Biosciences, San Jose, CA, USA; 50 µg/ml) to assess cell viability.

### Alizarin Red staining

Tissues were fixed immediately in 10% neutral-buffered formalin for 24 h before tissues were transferred to 70% EtOH. All further tissue processing and staining was performed by HistoTox Labs (Boulder, CO, USA). Tissues were paraffin embedded and sectioned (5 µm) by standard methods. Alizarin Red staining was performed to visualize regions of mineralization. An Olympus BX46 microscope (Center Valley, PA, USA) was used for all histological examinations. Images were captured utilizing an Olympus DP73 camera along with cellSens software for image acquisition. Sections were scored by a pathologist on a 0-4 scale (0, no finding; 1, minimal; 2, mild; 3, moderate; 4, marked) by blinded assessment. Mineralization was scored based on the staining intensity, size and frequency of mineralized foci. Images from 8-10 mice/group were used for scoring analysis.

### Statistical analyses

GraphPad Prism version 7.0 (GraphPad Software, San Diego, CA, USA) was used for statistical analyses. For normally distributed data, statistical analysis was performed using one-way ANOVA followed by Tukey's multiple comparison test. For data that were not normally distributed, Kruskal–Wallis nonparametric test followed by Dunn's multiple comparison test was performed. Statistical significance was defined as *P<0.05, **P<0.005 and ***P<0.0005, or as otherwise indicated.

## Supplementary Material

Supplementary information
