# Supplementary material for: ENPP1 enzyme replacement therapy improves blood pressure and cardiovascular function in a mouse model of generalized arterial calcification of infancy
Source: Dis Model Mech. 2018 Oct 8;11(10):dmm035691. doi: 10.1242/dmm.035691 (PMC6215426; doi:10.1242/dmm.035691)
Supplement: Supplementary information [file dmm-11-035691-s1.pdf]

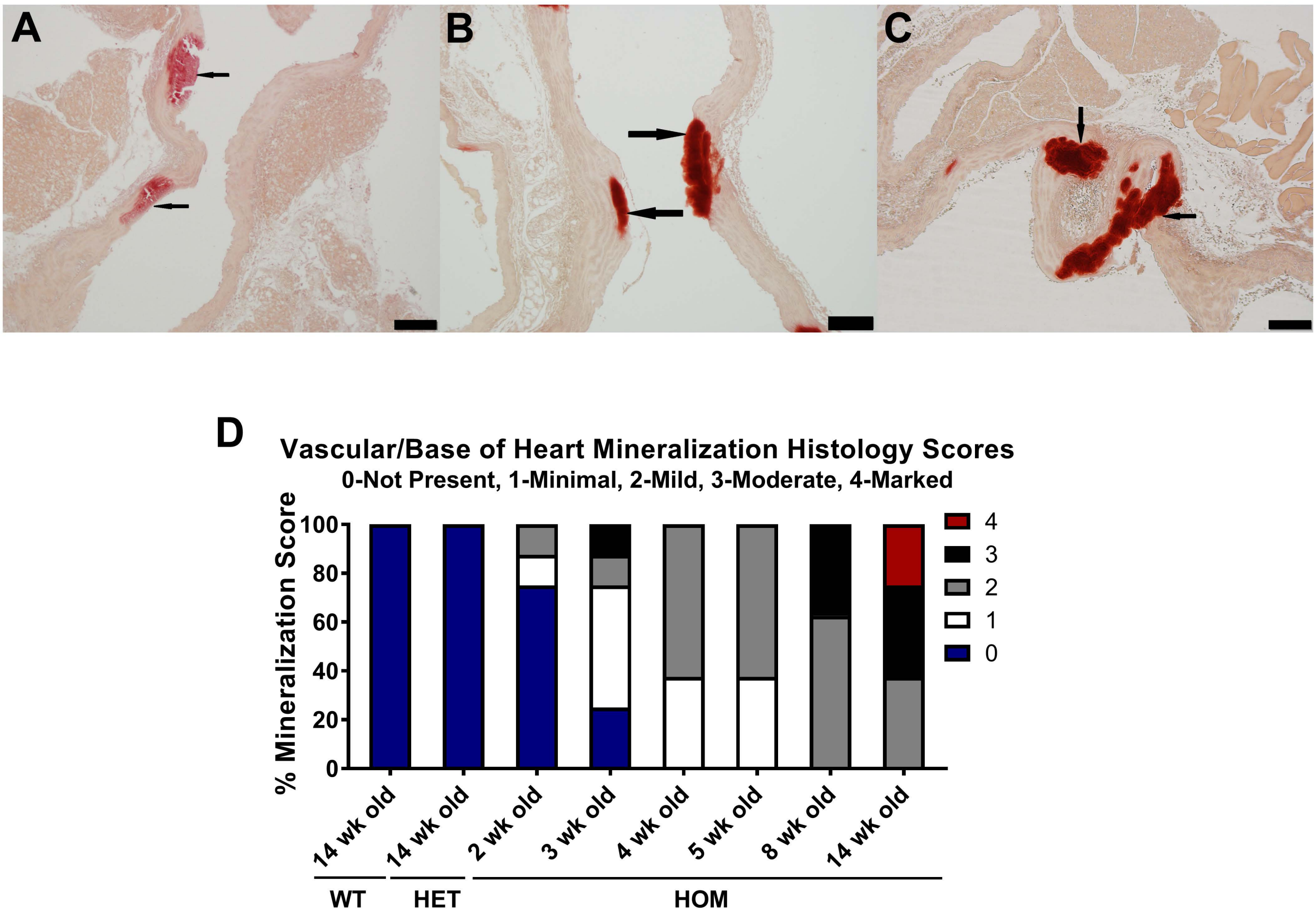


**Fig. S1. Alizarin Red staining of vascular/base of heart regions of *Asj-2J* mice displays areas of mineralization. (A-C)**: Representative Alizarin Red staining images from 5-week-old (score 2, mild) (A), 8-week-old (score 3, moderate) (B), and 14-week-old (score 4, marked) (C) *Asj-2J* hom mice. Areas of mineralization appear dark red (arrows). Scale bar represents 50 µm. **(D)** Vascular/base of heart mineralization histology scores for 2- to 14-week old mice (n=8 male mice/group). Severity of mineralization was graded on a 0- to 4- scoring scale based on the relative frequency, size, and staining intensity of the mineralized foci that were present. The bar graph shows the percentage of mice at each score level.

Disease Models & Mechanisms • Supplementary information

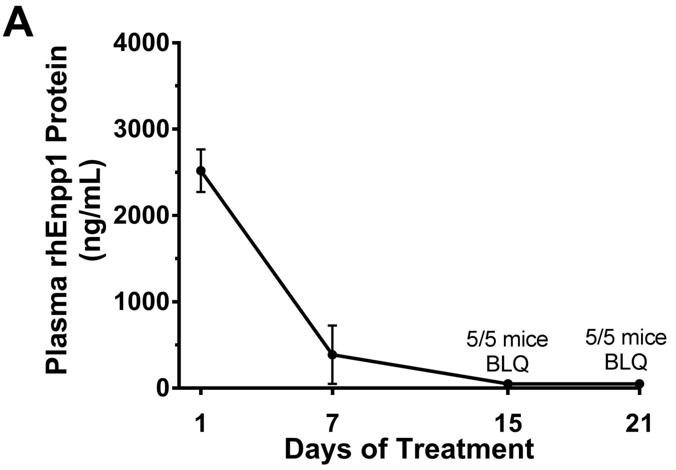


**Fig. S2. *Asj-2J* mice develop ADAs in response to rhENPP1 treatment.** Seven-week-old *Asj-2J* mice were treated with 5000 U/kg rhENPP1 (lot#1) or vehicle for a period of 3 weeks. Plasma rhENPP1 protein levels **(A)** and ADA levels **(B)** were monitored over the course of the study. BLQ= below the limit of quantitation. A cohort of 5 male mice was monitored in this study. All data is graphed as mean +/- S.E.M.

Disease Models & Mechanisms • Supplementary information

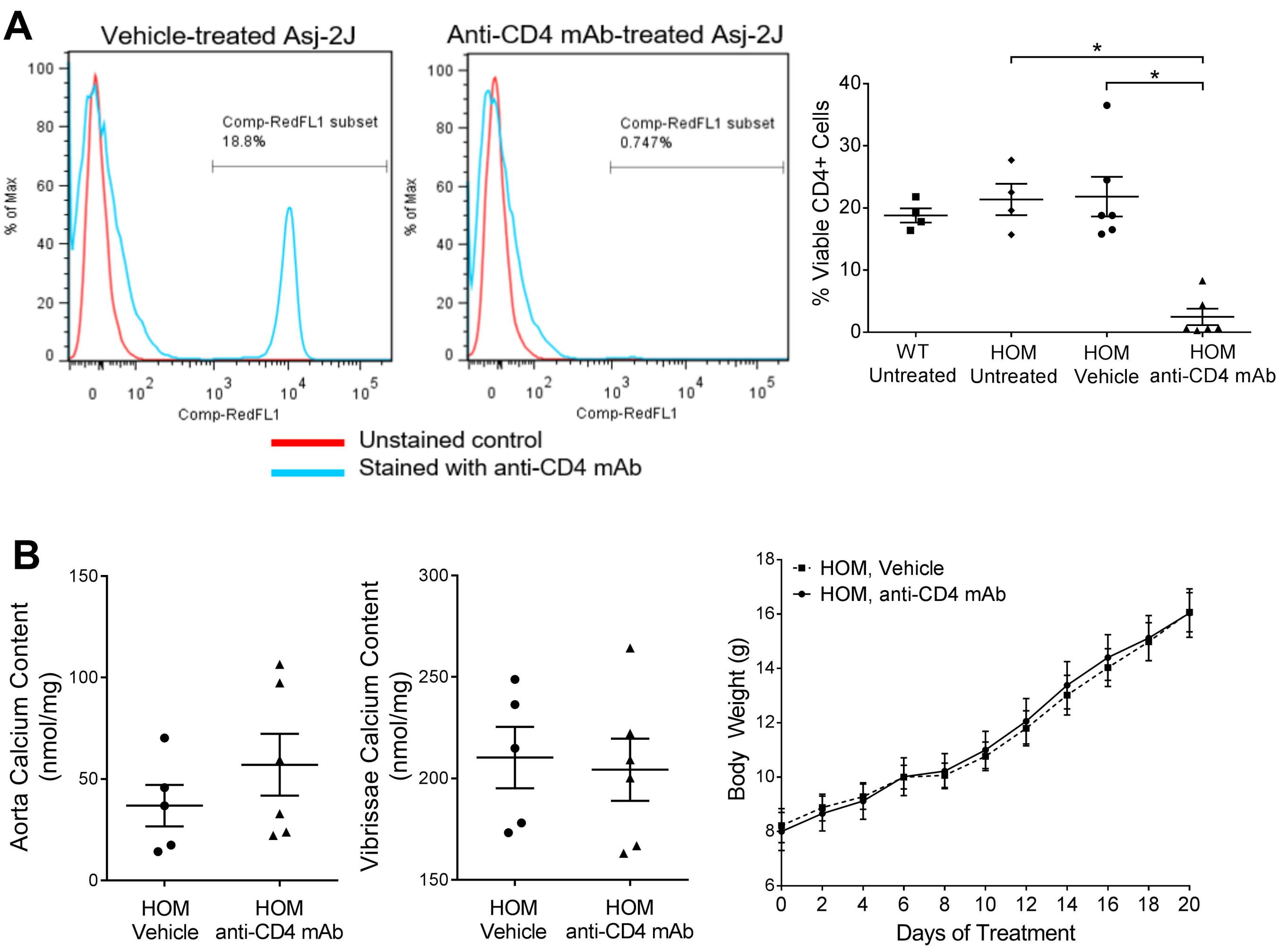


**Fig. S3. Anti-CD4 mAb treatment depletes CD4+ T cells by >90% in peripheral blood of *Asj-2J* mice. (A)** Representative flow cytometry plots showing the percentage of CD4+ T cells in the peripheral blood of 4-week-old *Asj-2J* mice one week after a single injection of vehicle (left) or 300 µg anti-CD4 mAb (middle). Quantitation of the results from all animals (right). Each treatment group consists of a cohort of n=4 (WT and untreated *Asj-2J*) or n=6 (vehicle- and anti-CD4-treated *Asj-2J*) male mice/group. * P < 0.03 (by Kruskal-Wallis test, followed by Dunn's multiple comparison test). **(B)** Calcification levels in aorta (left) and muzzle skin biopsies containing the dermal sheath of vibrissae (middle) of 5-week-old mice dosed with 300 µg of anti-CD4 mAb at 2 weeks of age and 100 µg at 3 and 4 weeks of age. Body weights of these mice was monitored (right). Each treatment group consists of a cohort of n=5 (vehicle-treated *Asj-2J*) or n=6 (anti-CD4-treated *Asj-2J*) male mice/group.All data is graphed as mean +/- S.E.M. All statistical analyses were performed relative to vehicle-treated *Asj-2J* hom mice. *P < 0.05, **P < 0.005, and *** P < 0.0005 (by one-way ANOVA, followed by Tukey's multiple comparison test).

Disease Models & Mechanisms • Supplementary information

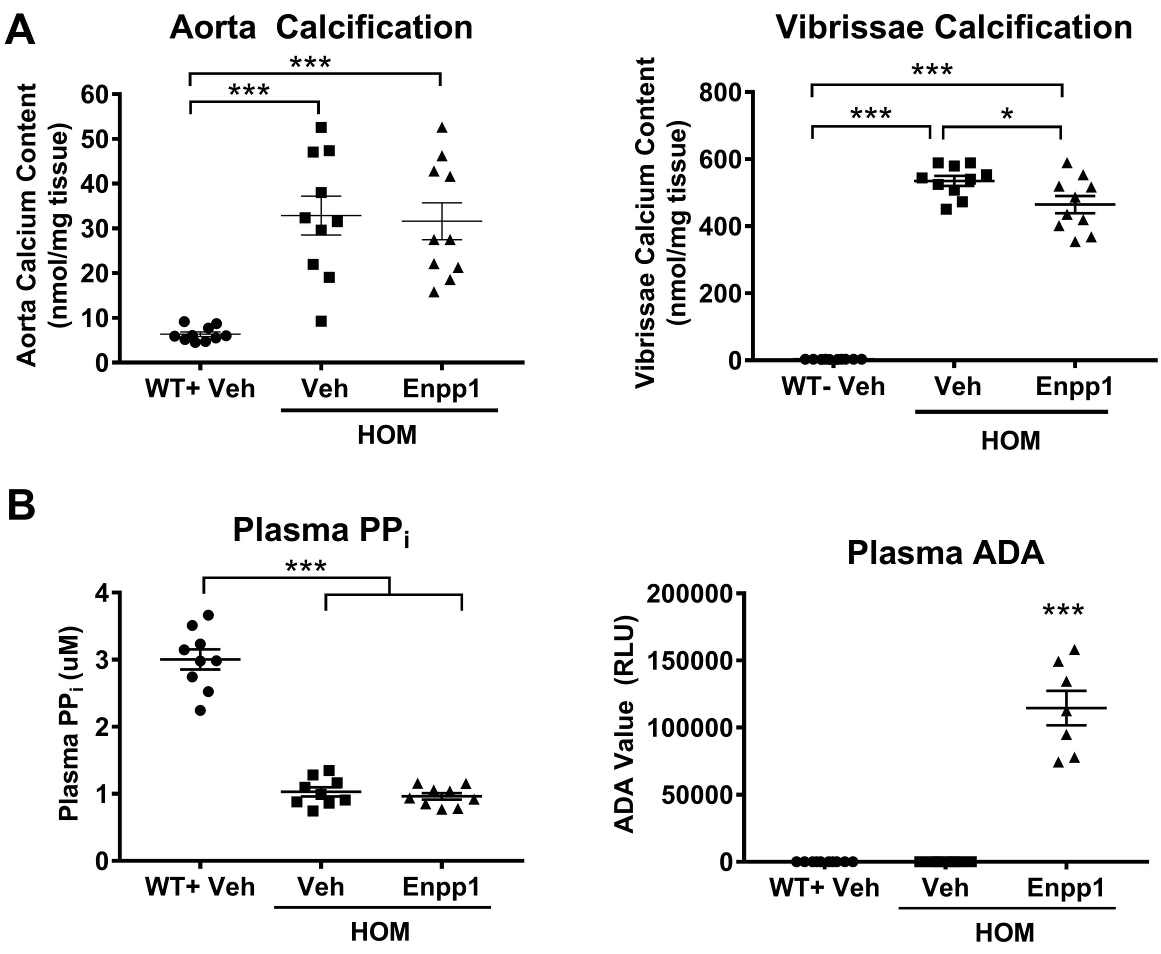


**Fig. S4. rhENPP1 treatment for 6 weeks does not prevent calcification development in *Asj-2J* mice.** Mice were dosed beginning at 2 week of age with 2100 U/kg rhENPP1 (lot #2) or vehicle EOD by subcutaneous injection for a period of 6 weeks. Tissues and plasma were collected 1 day after the final dose. **(A)** Calcification levels in aorta and muzzle skin containing the dermal sheath of vibrissae were measured using a quantitative calcium assay. **(B)** Plasma $PP_i$ and ADA levels were measured in mice. Each treatment group consists of a cohort of n=8 mice/group and all data is graphed as mean +/- S.E.M. *P < 0.05, **P < 0.005, and *** P < 0.0005 (by one-way ANOVA, followed by Tukey's multiple comparison test).

Disease Models & Mechanisms • Supplementary information
